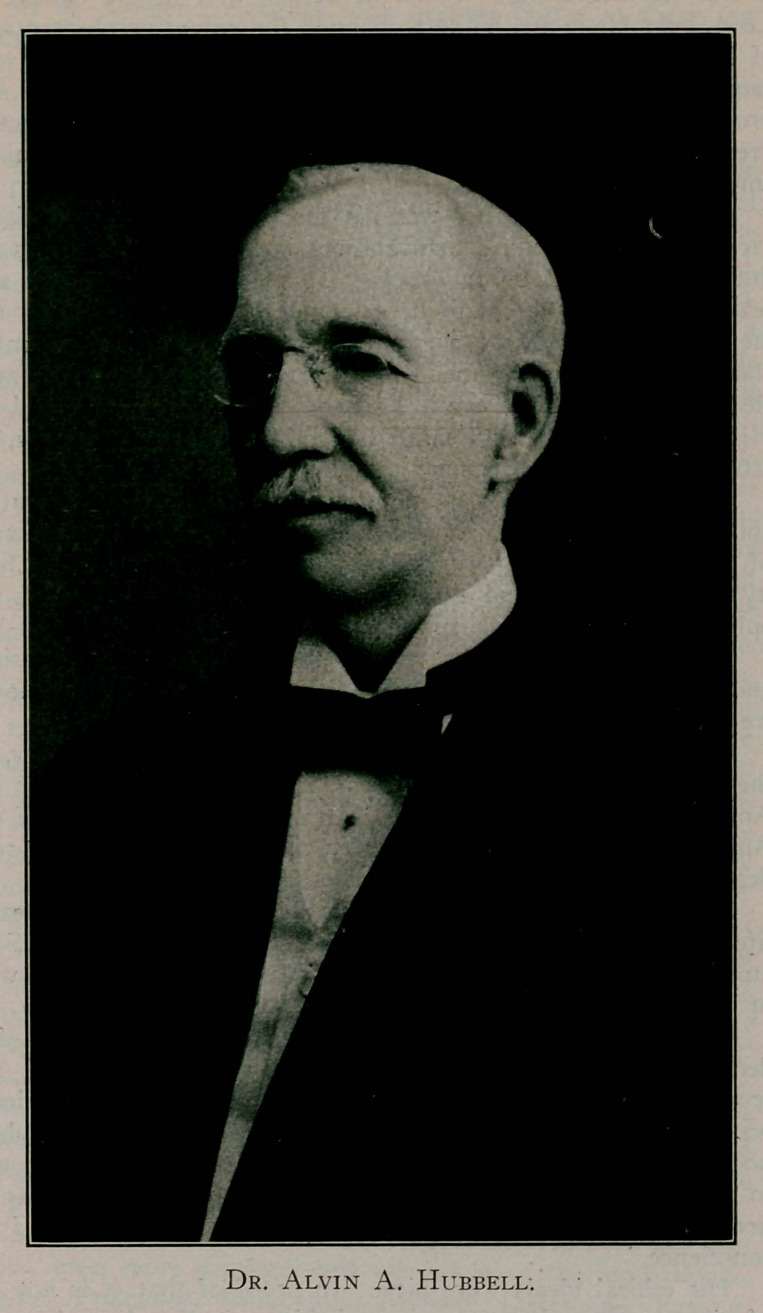# The Loss to the Profession in the Death of Dr. Alvin A. Hubbell

**Published:** 1911-09

**Authors:** 


### BUFFALO MEDICAL JOURNAL

**A Monthly Review of Medicine and Surgery**

*EDITOR*
A. L. BENEDICT.

All communications, whether of a literary or business nature, books for review and exchanges, should be addressed to the editor, 354 FRANKLIN STREET, BUFFALO, N. Y. Please make personal or telephonic calls before 1 p. m.

VOL. LXVII. SEPTEMBER, 1911. No. 2

### The Loss to the Profession in the death of Dr. Alvin A. Hubbell

IT has always been customary for the JOURNAL to publish obituary notices or sketches of the lives of medical men who have practised in Buffalo or in western New York; but only occasionally is a professional career so conspicuous in its success as to call for editorial comment. Such, however, was the case with Dr. Alvin A. Hubbell, who died at the Lenox Hotel on the 10th of August, 1911.

The daily papers at the time gave the leading facts concerning his life, and to detail them again would be useless repetition suited rather for the general public. It is proper to add, however, that for a number of years Dr. Hubbell was Associate Editor of the BUFFALO MEDICAL JOURNAL, and that particularly in his chosen field of specialism he added greatly to the interest and success of the magazine.

But when a practitioner has achieved a position of real eminence among us and then laid down his work, it is instructive to glance at the characteristics which made him what he was, and fitting also to pay some tribute of respect to his memory. Especially edifying is it to see how the simple, sturdy and homely virtues tell for success in medicine as in every other walk of life.

For Dr. Hubbell came from a race of pioneers, and almost alone he blazed his own path from a farm in the backwoods to a position of professional eminence which most of us might well envy.

Perhaps the foremost element in his character was an insatiable thirst for knowledge. Sixty-five years ago when he began life near the town of Conewango, Cattaragus County, the prospects for liberal education were small. But he outgrew the narrow confines, as an oak springs from the acorn. The district school, the local academy in Randolph, then more study, the illuminating example of a neighboring physician—all prepared him for courses at the University of Pennsylvania, and later at Buffalo, where he took his final degree in 1876.

Dr. Alvin A. Hubbell.

To some men, a diploma means the end of study. To him it was the beginning. When he settled first in the hamlet of Leon, near his home, he only changed the extensive observations of the university for others more intensive on the patients who came to him. In 1880 he came to Buffalo. Here, his ability and professional acumen were soon recognised. As his practice grew he studied the harder, and soon became a man of mark among ophthalmologists.

Another characteristic was his thorough appreciation of the fact that as the medical man absorbs knowledge, it becomes his duty to hand that on to others. This he did as a writer and teacher. It is unnecessary here to make a catalogue of his numerous articles. Most were short and practical, some more elaborate. His improvement of the smaller eye magnet, his articles on injuries of the eye in de Schweinitz and Randall and his History of the Earlier Years of Ophthalmology in America, all attracted more than ordinary attention.

Early in his career he developed rather unusual executive ability. He was one of those to organise the medical department of the Niagara University, one of the first surgeons of the Charity Eye, Ear, Nose and Throat Hospital and for many years the ophthalmologist of the hospital of the Sisters of Charity.

With such an untiring zeal in the study of his profession, and with such efficient results it is not strange that his merits were recognised by the profession.

He was elected in turn President of the County Society, of the State Association, of the Section of Ophthalmology of the American Medical Association. He was made a member of the American Ophthalmological Society, and was given an honorary degree of Doctor of Philosophy by Niagara University.

His domestic relations were unusually happy. In rather early life he married Miss Evangeline Fancher, a sister of former Senator Fancher. They had one daughter Bula, who is now the wife of Professor Everett Ward Olmsted of Cornell.

Dr. Hubbell's religious convictions were strong, and of a decided liberal trend. He was for several years one of the trustees of the Unitarian Church, and at the time of his death was a trustee of the social settlement known as the "Neighborhood House." He was impatient with cant and hypocrisy, and to quote a well known writer "he stands before his maker as an honest man, with no mask upon his face, and no shackles of his conscience."

His ethical ideals were high. In proof of that, it is not out of place to state that the writer of this notice has been for over thirty years his most constant and most prominent rival for professsional honors, and now at the end of the race, joins the host of his former colleagues with deepest regret in these few words of final and affectionate farewell.

## Figures and Tables

**Figure f1:**